# DT-010 Exerts Cardioprotective Effects by Regulating the Crosstalk between the AMPK/PGC-1*α* Pathway and ERp57

**DOI:** 10.1155/2023/8047752

**Published:** 2023-02-10

**Authors:** Xiaojing Zhang, Ximin Wu, Huihui Hu, Xiaoping Liu, Zhanfang Kang, Xin Deng

**Affiliations:** ^1^Department of Pharmacy, The Sixth Affiliated Hospital of Guangzhou Medical University, Qingyuan People's Hospital, Qingyuan, Guangdong, China; ^2^Institute of New Drug Research and Guangdong Province Key Laboratory of Pharmacodynamic Constituents of Traditional Chinese Medicine, College of Pharmacy, Jinan University, Guangzhou, Guangdong, China; ^3^Department of Basic Medical Research, The Sixth Affiliated Hospital of Guangzhou Medical University, Qingyuan People's Hospital, Qingyuan, Guangdong, China

## Abstract

The AMP-activated protein kinase (AMPK)/peroxisome proliferator-activated receptor *γ* coactivator 1*α* (PGC-1*α*) pathway performs a crucial role in energy metabolism and mitochondrial network. Our previous study found that DT-010, a novel danshensu (DSS) and tetramethylpyrazine (TMP) conjugate, had significant cardioprotective properties *in vitro* and *in vivo*. We also reported that ERp57 served as a major target of DSS using the chemical proteomics approach. In this article, we focus on exploring the interrelationship between the regulation of the AMPK/PGC-1*α* pathway and promoting ERp57 expression induced by DT-010 in *tert*-butylhydroperoxide- (t-BHP-) induced H9c2 cell injury. The results showed that DT-010 activated the AMPK/PGC-1*α* pathway and increased ERp57 protein expression. Importantly, the above phenomenon as well as the mitochondrial function can be partially reversed by siRNA-mediated ERp57 suppression. Meanwhile, silencing AMPK significantly inhibited the ERp57 expression induced by DT-010. In addition, molecular docking and kinase assay *in vitro* revealed that DT-010 had no direct regulation effects on AMPK activity. Taken together, DT-010 exerted cardioprotective effects by regulating the crosstalk of AMPK/PGC-1*α* pathway and ERp57, representing a potential therapeutic agent for ischemic heart disease.

## 1. Introduction

Ischemic heart disease (IHD) is the top leading cause of cardiovascular diseases (CVDs) health lost [1, 2]. Variable treatment plans are available depending on the symptoms and types of CVDs. The health status of the general population has improved dramatically over the past few decades, benefiting from lifestyle changes, medications, surgery, cardiac rehabilitation, and active surveillance [3]. However, there remains no effective therapeutic agent for preventing myocardial ischemia-reperfusion injury (MIRI) in patients; hence, new strategies and methods are in urgent need. MIRI leads to mitochondrial damage by affecting energy metabolism, mitochondrial-related inflammation, mitochondrial dynamics, etc. [4] Multiple studies have also shown that improving mitochondrial function significantly reduced MIRI [5–7]. Mitochondrial-targeted clinical intervention trials to restore mitochondrial function have been underway for many years, although there are still no mitochondrial-targeted drugs (MTDs) for the treatment of CVDs specifically [8–11]. Novel MTDs for IHD are still needed.

Accumulating studies have demonstrated a positive interconnectedness between the AMP-activated protein kinase (AMPK) and the mitochondrial net [12]. Mitochondrial dysfunction and oxidative stress mediated by AMPK deficiency are essential processes in MIRI. Peroxisome proliferator-activated receptor gamma coactivator-1 alpha (PGC-1*α*) is a key regulator of mitochondrial biogenesis [13], and it may contribute to enhanced mitochondrial DNA (mtDNA) copy number, an indicator of disease aggressiveness. Overexpression of PGC-1*α* improves I/R-induced cardiomyocyte apoptosis [14]. Activated PGC-1*α*/nuclear factor erythroid 2-related factor 2 (Nrf2) signaling exerted an antioxidant effect in oxygen-glucose deprivation/reperfusion- (OGD/R-) induced injury in H9c2 cells [15]. Emerging research has shown that the activation of the AMPK/PGC-1*α* pathway could decrease mitochondrial dysfunction and apoptosis [16]. Hence, activation of the AMPK/PGC-1*α* pathway may be one of the strategies for the treatment of IHD.

Our previous studies had shown that DT-010 exerted cardioprotective effects by activating the PGC-1*α*/Nrf2/heme oxygenase 1 (HO-1) pathway [17, 18]. However, the upstream regulator in terms of DT-010 is unclear. Furthermore, a chemical proteomics approach was used, and we identified endoplasmic reticulum (ER) resident protein 57 (ERp57) as a potential target of DT-010 [18]. ERp57 is an ER resident thiol-disulfide oxidoreductase and acts as a redox-dependent chaperone. Prolonged ER stress causes cellular dysfunction that leads to CVDs. The study found that cell viability and clonogenic survival are reduced upon the depletion of ERp57 [19]. However, the function of ERp57 in cardioprotection has rarely been studied. It is also unclear whether DT-010 acts majorly on the crosstalk between the AMPK/PGC-1*α* pathway and ERp57. In the following, we explored the hypothesis of DT-010 in a model of *tert*-butylhydroperoxide- (t-BHP-) injured H9c2 cells and its associated signal pathways. This finding will provide new insight into CVD treatment.

## 2. Materials and Methods

### 2.1. Chemicals and Reagents

DT-010 (purity > 98%) was obtained from Jinan University (Guangzhou, China). t-BHP, 3-(4,5-dimethylthiazol-2-yl)-2,5-diphenyl tetrazolium bromide (MTT), phenylmethylsulfonyl fluoride (PMSF), and phosphatase inhibitor were acquired from Sigma-Aldrich (St. Louis, MO, USA). ERp57 siRNA as well as the scrambled siRNA, antibodies against PGC-1*α* and ERp57, were purchased from Santa Cruz Biotechnology, Inc. (Delaware Avenue, CA, USA). Antibodies against AMPK*α*, phospho-AMPK*α* (Thr172), GAPDH, and horseradish peroxidase- (HRP-) conjugated anti-rabbit or mouse antibodies were from Cell Signaling Technology (Beverly, MA, USA). The Seahorse XF Cell Mito Stress Test Kit was obtained from Seahorse Bioscience (Billerica, MA, USA). Cell culture-related reagents were bought from Gibco (Grand Island, NY, USA). All other chemicals of analytical grade were purchased from other local sources.

### 2.2. Cell Culture and Treatment

The H9c2 cardiomyoblast cell line was cultured in DMEM containing 10% fetal bovine serum at 37°C in a 5% CO_2_ humidified incubator. After treatment, H9c2 cells were collected and processed for the MTT assay, cellular oxygen consumption measurement, and Western blot assays, respectively.

### 2.3. MTT Assay

For the cytotoxicity test, H9c2 cells were treated with DT-010 (10, 15, 20, 30, and 40 *μ*M) diluted in serum-free DMEM for 24 h or DT-010 (30 and 100 *μ*M) at different time points (1, 2, and 4 h). MTT (final concentration: 0.5 mg/mL) was then added and cultured for another 4 h. For the AMPK inhibitor test, H9c2 cells were preincubated with 1 mM compound C (an AMPK inhibitor) for 0.5 h. After discarding the medium, H9c2 cells were then treated with DT-010 (30 *μ*M) for 1 h, the supernatant was aspirated, and a fresh medium containing 150 *μ*M t-BHP was added for another 4 h. The microplate reader (BioTek Instruments, USA) was used to measure the absorbance at 570 nm. Cell viability (%) was calculated relative to the value of the group without any treatment.

### 2.4. Cellular Oxygen Consumption Measurement

Cellular oxygen consumption assay was performed according to the Agilent Cell Analysis Technology's protocol (Santa Clara, CA, USA). H9c2 cells were cultured in a Seahorse XF 24-well utility plate (1.5 × 10^4^ cells per well) for 24 h. H9c2 cells were then transfected with ERp57 siRNA or scrambled siRNA (40 pmols siRNA) for 48 h according to Lipofectamine™ 3000 Reagent Protocol. The transfected H9c2 cells were nurtured with DT-010 (30 *μ*M) for 2 h and followed by exposure to 150 *μ*M t-BHP for another 4 h. The oxygen consumption rate (OCR) parameter value was analyzed after the stimulation of oligomycin, FCCP, and rotenone plus antimycin A.

### 2.5. Western Blot Analysis

H9c2 cells were lysed with 30 *μ*L RIPA buffer containing 1 mM PMSF and 1% (*v*/*v*) phosphatase inhibitor on ice. The cell lysis supernatant was collected and quantified according to the procedure of the BCA protein assay kit (Thermo Fisher Scientific, MA, USA). SDS-PAGE (8%-15%) was used to separate proteins of different molecular weights. The polyvinylidene difluoride (PVDF) membranes were blocked with 5% nonfat dried milk. The blocking solution was removed, and specific primary antibodies against AMPK, pho-AMPK, ERp57, PGC-1*α*, and GAPDH (loading control) were added and incubated (all used at 1 : 1000 dilution) overnight at 4°C. HRP-conjugated antibody (1 : 2000) was added to the membranes for 2 h at room temperature. Western blot images and the densities of the bands were performed using the Bio-Rad Gel Imaging Systems (Bio-Rad, Hercules, CA, USA).

### 2.6. Molecular Docking

The 3D crystal structure of AMPK alpha-2 in composite with coumarin ADP was retrieved from the RCSB Protein Data Bank (PDB ID: 2H6D, Resolution: 1.85 Å). The molecular docking assay was performed using AutoDock Vina 1.2.2 software. The exhaustiveness is 8 and the dimensions of the grid box are *X*: 62, *Y*: 56, and *Z*: 50. The AMPK and DT-010 binding free energy was expressed in *Δ*Gb (kcal/mol). BIOVIA Discovery Studio Visualizer 4.5 was used to visualize the best-scored conformation of DT-010 and graphical representations.

### 2.7. AMPK Kinase Assay

This assay was conducted as stated in the previous report [20]. The reaction mixture (50 *μ*L) was conducted at 30°C for 40 min. The kinase activity was measured by quantitating the amount of ATP remaining in the solution using the Kinase-Glo Plus luminescence kinase assay kit. The IC_50_ values were calculated using GraphPad Prism software (nonlinear regression with normalized dose-response fit).

### 2.8. Data Analysis and Statistics

All data were processed and analyzed with GraphPad Prism software 9.0 (GraphPad, San Diego, CA, USA). Results were stated as mean ± standard deviation (mean ± SD). One-way ANOVA or two-way ANOVA and Dunnett's or Tukey's multiple comparison tests were assessed for comparison between groups. Differences with *P* < 0.05 were defined as significant.

## 3. Results

### 3.1. Cytotoxicity Study of DT-010

**Figure 1:**
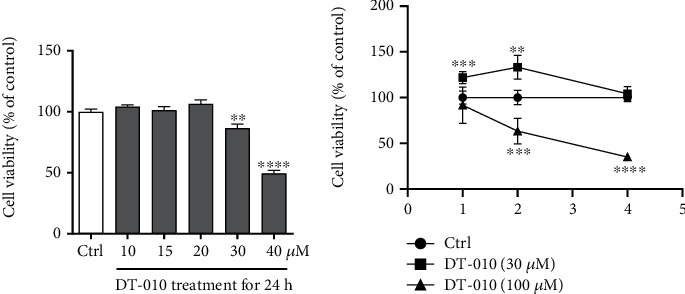
Effect of DT-010 on cell viability in H9c2 cells. (a, b) Cell viability (% of Ctrl) was measured by the MTT assay. ^∗∗^*P* < 0.01, ^∗∗∗^*P* < 0.001, and ^∗∗∗∗^*P* < 0.0001 versus the Ctrl group. Data are mean ± SD of three independent experiments. Data were analyzed using one-way ANOVA or two-way ANOVA and Dunnett's multiple comparison tests.

The cytotoxicity of DT-010 in H9c2 cells was assessed using MTT assay. As shown in Figure 1(a), DT-010 started to demonstrate cytotoxicity at 30 *μ*M. Next, we detected the cytotoxicity of DT-010 (30 and 100 *μ*M) at 1, 2, and 4 h in H9c2 cells. As shown in Figure 1(b), DT-010 (30 *μ*M) significantly promoted mitochondrial proliferation at 1 and 2 h. DT-010 was not cytotoxic at the 4 h time point. However, DT-010 (100 *μ*M) significantly reduced cell viability at 2 h and 4 h time points.

### 3.2. DT-010 Upregulated the p-AMPK, PGC-1*α*, and ERp57 Expressions

**Figure 2:**
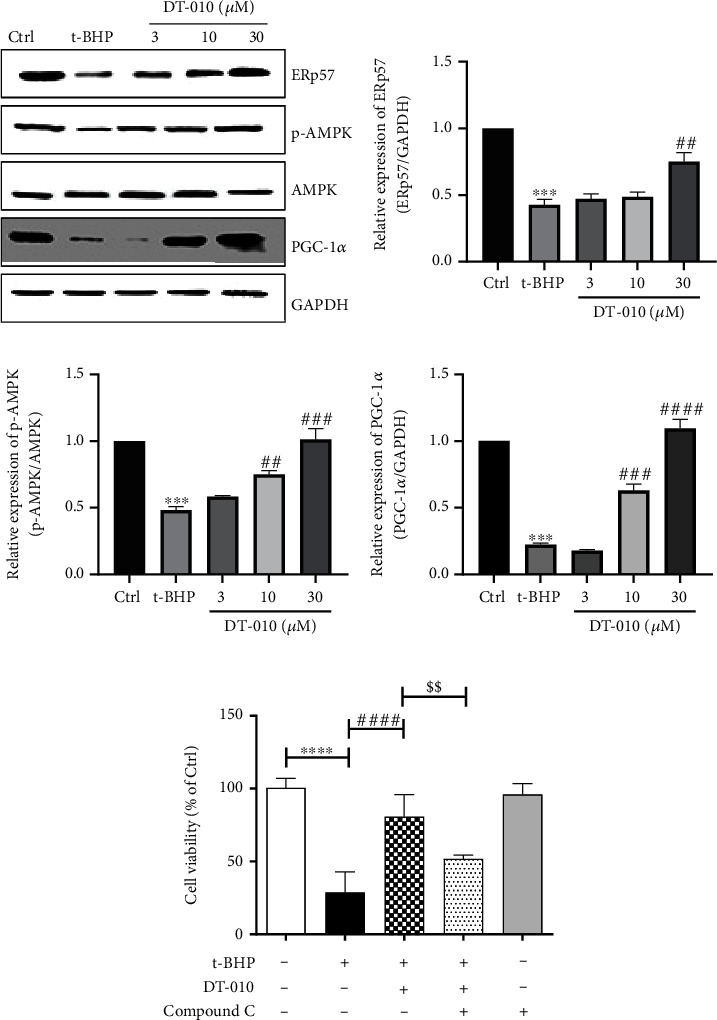
Effect of DT-010 on AMPK/PGC-1*α* pathway and ERp57 in H9c2 cells. (a–d) DT-010 increased the ERp57 (b), p-AMPK*α* (c), and PGC-1*α* (d) protein expression levels in t-BHP-induced H9c2 cell injury. (e) Cell viability (% of Ctrl) was measured by the MTT assay. Data are mean ± SD of two or three independent experiments. ^∗∗∗^*P* < 0.001 and ^∗∗∗∗^*P* < 0.001 versus the Ctrl group; ^##^*P* < 0.01, ^###^*P* < 0.001, and ^####^*P* < 0.0001 versus the t-BHP group. ^$$^*P* < 0.01. Data were analyzed using one-way ANOVA and Dunnett's multiple comparison test (b–d) or Tukey's multiple comparison tests (e).

Immunoblotting showed that the ERp57, p-AMPK, and PGC-1*α* protein expressions in the t-BHP group were reduced, and DT-010 administration eliminated these differences (Figures 2(a)–2(d)). The above results demonstrated that the cardioprotective effect of DT-010 may be interconnected with the revision of ERp57 and the AMPK/PGC-1*α* signaling pathway. Compound C, also named dorsomorphin, is a potent, reversible, selective AMPK inhibitor. As shown in Figure 2(e), the cell viability was significantly decreased when AMPK activity was blocked (*P* < 0.01).

### 3.3. DT-010 Affects Mitochondrial Respiration Function by Regulating ERp57

**Figure 3:**
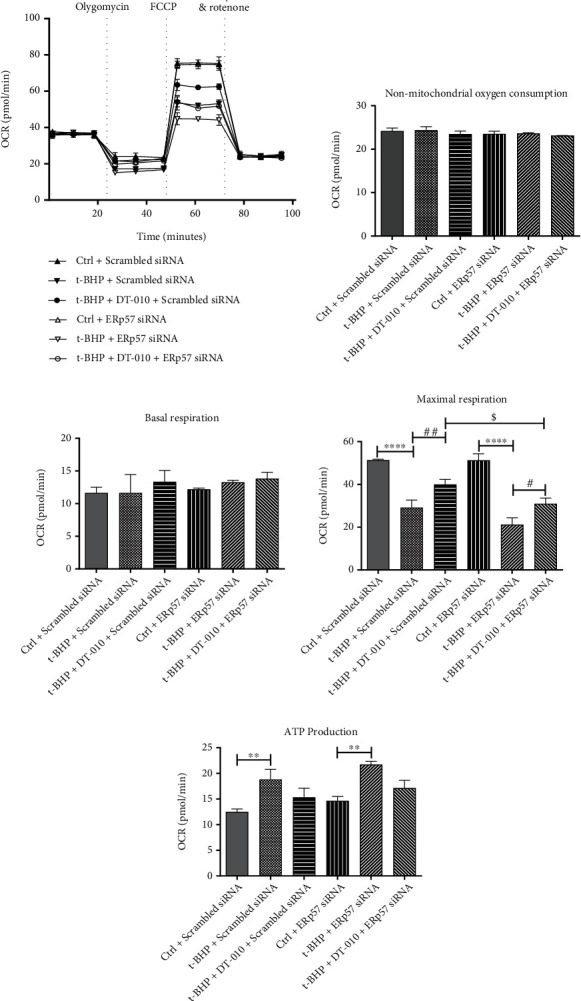
Effect of DT-010 on H9c2 cell mitochondrial respiratory function. (a) Representative oxygen consumption traces in H9c2 cells exposed sequentially to 1 *μ*M oligomycin, 0.5 *μ*M FCCP, and 1 *μ*M rotenone plus 1 *μ*M antimycin A. (b) Nonmitochondrial oxygen consumption. (c) Basal and (d) maximal respiration analysis. (e) ATP production. Data are mean ± SD of two or three independent experiments. ^∗∗^*P* < 0.01 and ^∗∗∗∗^*P* < 0.0001; ^#^*P* < 0.05 and ^##^*P* < 0.01; ^$^*P* < 0.05. Data were analyzed using one-way ANOVA and Tukey's multiple comparison tests.

OCR is a key indicator for mitochondrial respiration function and ATP production rate. To further verify the role of ERp57 on mitochondrial energy metabolism, the OCR assay was evaluated using a Seahorse analyzer (Figure 3(a)). DT-010 did not alter nonmitochondrial oxygen consumption (Figure 3(b)) and basal respiration (Figure 3(c)) when ERp57 was blocked. However, DT-010 significantly enhanced the maximal OCR (Figure 3(d), *P* < 0.01) in H9c2 cells and decreased the ATP production (Figure 3(e)). Compared with the t-BHP+DT-010+scrambled siRNA group, silencing the expression of ERp57 reduced the effect of DT-010 on ameliorating the above changes. Altogether, these results showed that DT-010 could enhance mitochondrial respiration function and maintain homeostasis by regulating ERp57 expression.

### 3.4. Upregulation of ERp57 Expression by DT-010 Depends on AMPK Activation

**Figure 4:**
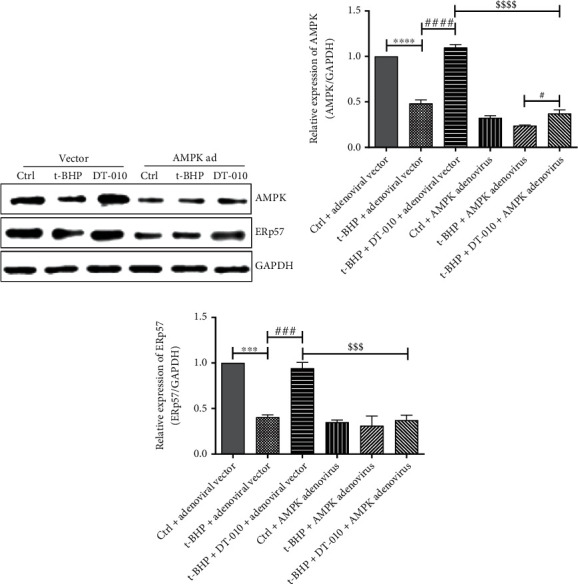
Adenovirus-mediated knockdown of AMPK abolished the upregulation of DT-010 on ERp57 protein expression in H9c2 cells. (a) Representative Western blot of AMPK and ERp57. (b, c) Quantification of Western blot band density of AMPK (b) and ERp57 (c). Data are mean ± SD of two independent experiments. ^∗∗∗^*P* < 0.001 and ^∗∗∗∗^*P* < 0.0001; ^#^*P* < 0.05, ^###^*P* < 0.001, and ^####^*P* < 0.0001; ^$$$^*P* < 0.001 and ^$$$$^*P* < 0.0001. Data were analyzed using one-way ANOVA and Tukey's multiple comparison tests.

Our results confirmed that DT-010 can promote AMPK phosphorylation and upregulate ERp57 protein expression; thus, the upstream and downstream regulatory relationship of DT-010 on AMPK and ERp57 was determined. As shown in Figure 4, compared with the DT-010+adenovirus vector group, the expression of ERp57 protein was significantly decreased after the knockdown of AMPK mediated by the adenovirus. The results indicated that DT-010 may regulate the expression of ERp57 by activating AMPK, thereby affecting mitochondrial function.

### 3.5. Upregulation of PGC-1*α* Expression by DT-010 Is Dependent on ERp57 Activation

**Figure 5:**
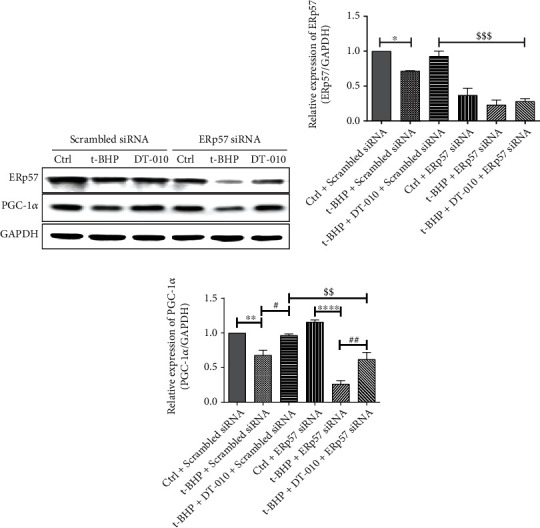
Knockdown ERp57 by specific shRNA abolished the upregulation of DT-010 on PGC-1*α* protein expression in H9c2 cells. (a) Representative Western blot of ERp57 and PGC-1*α*. (b, c) Quantification of Western blot band density of ERp57 (b) and PGC-1*α* (c). Data are mean ± SD of two independent experiments. ^∗^*P* < 0.05, ^∗∗^*P* < 0.01, and ^∗∗∗∗^*P* < 0.0001; ^#^*P* < 0.05 and ^##^*P* < 0.01; ^$$^*P* < 0.01 and ^$$$^*P* < 0.001. Data were analyzed using one-way ANOVA and Tukey's multiple comparison tests.

To further verify the upstream and downstream relationship of DT-010 referring to ERp57 and PGC-1*α* expression, we next evaluated the influence of DT-010 on PGC-1*α* protein expression by specific shRNA of ERp57. As revealed in Figure 5, DT-010 significantly increased the expression of ERp57 and PGC-1*α* when matched to the t-BHP+scrambled siRNA group (*P* < 0.01). Compared with the t-BHP+DT-010+scrambled siRNA group, the upregulation trend of PGC-1*α* was partially abolished by ERp57-specific shRNA. The result reflected that DT-010 may regulate PGC-1*α* expression by regulating ERp57 expression.

### 3.6. DT-010 Has No Direct Regulatory Effect on AMPK Kinase

**Figure 6:**
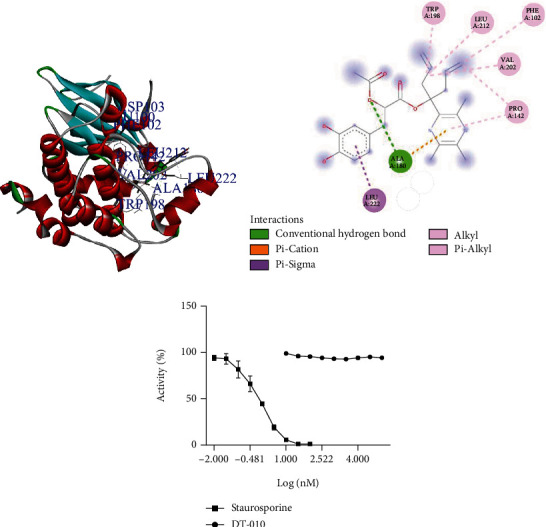
Molecular docking simulation of interaction and AMPK kinase assay. (a) Molecular visualization of the interaction between DT-010 and the target human protein AMPK. (b) The interaction of amino acid residues in AMPK with DT-010. (c) AMPK activity (%).

As shown in Figures 6(a) and 6(b), DT-010 could dock into the active site of AMPK, with an average binding free energy of -6.6 kcal/mol. The major interactions between DT-010 and the amino acid residues were Ala-180, Pro-142, Val-202, Trp-198, Leu-212, Phe-102, and Leu-222, however, via weak hydrogen bonds. *In vitro* kinase assay further demonstrated that DT-010 did not affect AMPK activity (Figure 6(c)). The results suggested that DT-010 has no direct effect on AMPK.

## 4. Discussion

IHD remains a major disease with an increasing overall global burden. Thrombolysis and percutaneous coronary intervention are effective and are extensively applied, which can restore myocardial perfusion timely and alleviate myocardial infarction or necrosis [21]. Myocardial ischemic reperfusion injury (MIRI) that comes along with restoring blood flow may further worsen the prognosis in these patients. According to the statistics of the World Health Organization, one out of every six deaths was attributed to MIRI [22]. Research is being carried on; however, there is still no effective therapeutic strategy to prevent MIRI [21]. Mitochondrial dysfunction is involved in the pathological process of myocardial infarction. The study found that impaired mitochondrial biosynthesis was the common cause of end-stage ischemic heart failure and cardiac hypertrophy [12]. Multiple clinical studies have found that mitochondrial targeting agents (MTAs) have limited benefit in the treatment of CVD, properly due to the poor uptake by ischemic tissue, the single regulatory target, the inefficient mitochondrial localization or tissue-specific delivery, etc. [23] Therefore, targeting mitochondria from multiple angles is still an important treatment strategy for CVDs, and efforts should be made to improve the clinical transformation rate [24, 25]. In our previous study, we found that DT-010 exhibited brilliant antioxidant and antiapoptotic properties which were at least partially concerned with the activation of the PGC-1*α*/Nrf2/HO-1 pathway [17]. Continuing in-depth studies, we revealed that DT-010 displayed cardioprotective effects by regulating the axis of AMPK/PGC-1*α* pathway signaling and ERp57 crosstalk. Our results suggest that DT-010 maybe a promising MTA for myocardial ischemia therapy.

Overexpression of AMPK improved mitochondrial biosynthesis by maintaining mitochondrial redox homeostasis and mitochondrial respiration and inhibiting mitochondrial apoptosis [26]. The PGC-1*α* pathway was downregulated in rat and mouse models of heart failure [27]. Notably, PGC-1*α* transcription was blocked in heart failure caused by myocardial infarction [28]. Meanwhile, mitochondrial number and mtDNA copy number were reduced in rodent and human models of heart failure. The study found that AMPK could improve cardiac contractility after myocardial infarction by promoting PGC-1*α* expression and enhancing mitochondrial respiration [29]. AMPK/PGC-1*α* pathway activation prevented acute cardiotoxicity by maintaining mitochondrial homeostasis [30]. Therefore, targeting the AMPK/PGC-1*α* pathway might be one of the important strategies for the treatment of CVDs. Our results showed that DT-010 increased the expression of the AMPK/PGC-1*α* pathway. Importantly, the cardioprotective effects were reversed by AMPK inhibitor compound C *in vitro*. Interestingly, molecular docking and *in vitro* kinase experiments showed that DT-010 had no direct regulatory effect on AMPK. AMPK is widely expressed *in vivo* and regulates a variety of life activities, and AMPK heterodimeric complexes in different tissue subunit compositions vary widely, which may lead to reduced efficacy and increased drug toxicity. Although AMPK agonists have shown cardioprotective effects in ischemia-reperfusion mouse model. Proper regulation of AMPK should exert its protective potential while avoiding its potentially harmful effects. We speculated that DT-010 was superior to these AMPK activators as DT-010 not only had a significant cardioprotective effect by activating the AMPK/PGC-1*α* pathway but also had a high safety profile.

Studies have found that ERp57 was involved in a range of disease processes, including neurological disease [31], cancer [32], and infection diseases [33]. However, it is unclear the association between ERp57 and cardioprotection. ERp57/PDIA3 overexpression inhibited the ER stress and apoptosis induced by the SOD1 mutant, suggesting ERp57's role in neuroprotection [34, 35]. Wang et al. demonstrated that ERp57/PDIA3 deficiency reduced inflammation, oxidative pressure, and apoptosis in traumatic brain injury (TBI) mice [36], indicating that ERp57/PDIA3 had different roles in different cellular processes in different cellular compartments. ERp57/PDIA3 might also participate in the modulation of mitochondrial Ca^2+^ indirectly through the interaction with calnexin [37]. The t-BHP-mediated intracellular Ca^2+^ increase could induce ROS generation and mitochondrial dysfunction [38]. Our previous study has found that DT-010 could reduce t-BHP-induced intracellular free radical production, including hydroxyl radical (·OH), superoxide anion(·O^2−^), and peroxynitrite radical (ONOO^−^). Moreover, DT-010 triggered Nrf2 nuclear translocation and upregulated the protein expression of Nrf2 as well as mitochondrial transcription factor A (Tfam) and HO-1 in H9c2 cells [17]. We speculated that DT-010 may affect mitochondrial calcium homeostasis by regulating ERp57 to control ROS generation and mitochondrial function. Our published literature revealed that ERp57 was a major target of biotinylated danshensu (DSS) using the chemical proteomics approach and showed that ADTM, a novel DSS and tetramethylpyrazine (TMP) conjugate, exhibited potent inhibition on the redox activity of ERp57/PDIA3 [18]. Based on the structural similarity between ADTM and DT-010, we reasoned that DT-010 might have a regulatory effect on ERp57. We next explored the relationship between the regulation of the AMPK/PGC-1*α* pathway and promoting ERp57 expression induced by DT-010 in H9c2 cell oxidative injury model. The results showed that DT-010 activated the AMPK/PGC-1*α* pathway and increased the ERp57 protein expression. Importantly, specific siRNA-mediated ERp57 suppression partially reduced the PGC-1*α* protein expression and inhibited the mitochondrial functions of DT-010. Meanwhile, silencing AMPK significantly inhibited the expression of ERp57 of DT-010.

## 5. Conclusion

We speculated that DT-010 may have a different mode of action from that of ADTM. We found a feedback loop regulated by DT-010 among AMPK, PGC-1*α*, and ERp57, which are associated with oxidative stress and mitochondrial dysfunction. Our bountiful research work exhibited enormous promise for its involvement in CVDs.

## Data Availability

The raw data supporting the conclusion of this article will be made available by the corresponding authors.
